# Draft Genome Sequence of Streptococcus anginosus Strain CALM001, Isolated from the Gut of an Elderly Dane

**DOI:** 10.1128/MRA.00379-19

**Published:** 2019-06-13

**Authors:** Hajar Fauzan Ahmad, Lars Schreiber, Ian P. G. Marshall, Philip Junker Andersen, Josué Leonardo Castro-Mejía, Dennis Sandris Nielsen

**Affiliations:** a Department of Food Science, University of Copenhagen, Copenhagen, Denmark; b Center for Geomicrobiology (CfG), Aarhus University, Aarhus, Denmark; c Faculty of Industrial Science and Technology, Universiti Malaysia Pahang, Pekan, Pahang, Malaysia; d Energy, Mining and Environment, National Research Council, Montreal, Quebec, Canada; Loyola University Chicago

## Abstract

Here, we present a 1.89-Mbp draft genome sequence of Streptococcus anginosus strain CALM001, a Gram-positive bacterium that was isolated from a fecal sample donated by a 70-year-old Dane enrolled in the Counteracting Age-Related Loss of Skeletal Muscle Mass (CALM) intervention study.

## ANNOUNCEMENT

Streptococcus anginosus is a member of the “*Streptococcus milleri*” group (SMG), together with 2 other distinct species, Streptococcus intermedius and Streptococcus constellatus (1). Oral cavity, urogenital, and gastrointestinal specimens are the most common samples from which these streptococcal species have been recovered (2). Identification of the species from this group is difficult, as they share similar phenotypic characteristics (3).

S. anginosus strain CALM001 was isolated from the slurry of a homogenized fecal sample collected from the cohort of Counteracting Age-Related Loss of Skeletal Muscle Mass (CALM) intervention study (4) by overnight cultivation on Gifu anaerobic medium (GAM broth; HyServe GmbH & Co. KG). The genomic DNA of the strain was extracted using the GenElute bacterial genomic DNA kit (Sigma-Aldrich Co., St. Louis, MO). A sequencing library was prepared using the Nextera XT kit (Illumina, San Diego, CA). Genome sequencing was performed using the MiSeq platform (Illumina) with a paired-end 2 MiSeq reagent kit v2. The resulting ca. 1.97 million read pairs (≈0.88 Gbp) were inspected using FastQC v0.11.4 (http://www.bioinformatics.babraham.ac.uk/projects/fastqc/). Adapters and low-quality regions were trimmed using Trimmomatic v0.33 (5), with the following parameters: CROP:225 HEADCROP:20 SLIDINGWINDOW:4:20 ILLUMINACLIP:adapters.fasta:2:40:15 MINLEN:100. Trimmed reads were assembled using SPAdes v3.6.1 (6), with the following parameters: –k 21,33,55,77,99,127 –careful. Contigs that most likely originated from contamination were removed based on G+C content and coverage using MetaWatt v3.5 (7), reducing the assembly to contigs classified as originating from the genus Streptococcus. Trimmed reads mapping to the retained *Streptococcus* contigs (ca. 1.28 million read pairs, ≈0.48 Gbp) were extracted using BBMap v34.94 (https://sourceforge.net/projects/bbmap/) using the “minid=0.98” option.

The extracted reads were assembled using SPAdes, as described before, which resulted in eight contigs with ca. 254-fold coverage. The draft genome sequence of CALM001 has a total length of 1,888,139 bp, an average G+C content of 38.75%, and an *N*_50_ length of 1,134,963 bp. Using a marker gene set for the genus *Streptococcus*, CheckM v1.0.3 (8) estimates the genome to be 100% complete.

### Data availability.

The draft genome sequence of Streptococcus anginosus strain CALM001 is available in the GenBank database under accession number QWDL00000000. Raw sequencing data have been deposited in the SRA database under accession number SRX5707091, with BioSample and BioProject accession numbers SAMN09908147 and PRJNA487690, respectively.

## References

[1] Clarridge JE III, Attorri S, Musher DM, Hebert J, Dunbar S 2001 *Streptococcus intermedius*, *Streptococcus constellatus*, and *Streptococcus anginosus* (“*Streptococcus milleri* group”) are of different clinical importance and are not equally associated with abscess. Clin Infect Dis 32:1511–1515. doi:10.1086/320163. 11317256

[2] Rahman M, Nguyen SV, McCullor KA, King CJ, Jorgensen JH, McShan WM 2015 Complete genome sequence of *Streptococcus anginosus* J4211, a clinical isolate. Genome Announc 3:e01440-15. doi:10.1128/genomeA.01440-15. PMC4683221 26679576

[3] Clarridge JE III, Osting C, Jalali M, Osborne J, Waddington M 1999 Genotypic and phenotypic characterization of “*Streptococcus milleri*” group isolates from a Veterans Administration hospital population. J Clin Microbiol 37:3681–3687. 10523574 10.1128/jcm.37.11.3681-3687.1999 PMC85724

[4] Bechshøft RL, Reitelseder S, Højfeldt G, Castro-Mejía JL, Khakimov B, Bin Ahmad HF, Kjær M, Engelsen SB, Johansen SMB, Rasmussen MA, Lassen AJ, Jensen T, Beyer N, Serena A, Perez-Cueto FJA, Nielsen DS, Jespersen AP, Holm L 2016 Counteracting Age-Related Loss of Skeletal Muscle Mass: a clinical and ethnological trial on the role of protein supplementation and training load (CALM intervention study): study protocol for a randomized controlled trial. Trials 17:397. doi:10.1186/s13063-016-1512-0. 27507236 PMC4977774

[5] Bolger AM, Lohse M, Usadel B 2014 Trimmomatic: a flexible trimmer for Illumina sequence data. Bioinformatics 30:2114–2120. doi:10.1093/bioinformatics/btu170. 24695404 PMC4103590

[6] Bankevich A, Nurk S, Antipov D, Gurevich AA, Dvorkin M, Kulikov AS, Lesin VM, Nikolenko SI, Pham S, Prjibelski AD, Pyshkin AV, Sirotkin AV, Vyahhi N, Tesler G, Alekseyev MA, Pevzner PA 2012 SPAdes: a new genome assembly algorithm and its applications to single-cell sequencing. J Comput Biol 19:455–477. doi:10.1089/cmb.2012.0021. 22506599 PMC3342519

[7] Strous M, Kraft B, Bisdorf R, Tegetmeyer H 2012 The binning of metagenomic contigs for microbial physiology of mixed cultures. Front Microbiol 3:410. doi:10.3389/fmicb.2012.00410. 23227024 PMC3514610

[8] Parks DH, Imelfort M, Skennerton CT, Hugenholtz P, Tyson GW 2015 CheckM: assessing the quality of microbial genomes recovered from isolates, single cells, and metagenomes. Genome Res 25:1043–1055. doi:10.1101/gr.186072.114. 25977477 PMC4484387

